# Extension of Yeast Chronological Lifespan by Methylamine

**DOI:** 10.1371/journal.pone.0048982

**Published:** 2012-11-02

**Authors:** Sanjeev Kumar, Sophie D. Lefevre, Marten Veenhuis, Ida J. van der Klei

**Affiliations:** Molecular Cell Biology, Groningen Biomolecular Sciences and Biotechnology Institute (GBB), University of Groningen, Kluyver Centre for Genomics of Industrial Fermentation, Groningen, The Netherlands; Université Joseph Fourier, France

## Abstract

**Background:**

Chronological aging of yeast cells is commonly used as a model for aging of human post-mitotic cells. The yeast *Saccharomyces cerevisiae* grown on glucose in the presence of ammonium sulphate is mainly used in yeast aging research. We have analyzed chronological aging of the yeast *Hansenula polymorpha* grown at conditions that require primary peroxisome metabolism for growth.

**Methodology/Principal Findings:**

The chronological lifespan of *H. polymorpha* is strongly enhanced when cells are grown on methanol or ethanol, metabolized by peroxisome enzymes, relative to growth on glucose that does not require peroxisomes. The short lifespan of *H. polymorpha* on glucose is mainly due to medium acidification, whereas most likely ROS do not play an important role. Growth of cells on methanol/methylamine instead of methanol/ammonium sulphate resulted in further lifespan enhancement. This was unrelated to medium acidification. We show that oxidation of methylamine by peroxisomal amine oxidase at carbon starvation conditions is responsible for lifespan extension. The methylamine oxidation product formaldehyde is further oxidized resulting in NADH generation, which contributes to increased ATP generation and reduction of ROS levels in the stationary phase.

**Conclusion/Significance:**

We conclude that primary peroxisome metabolism enhanced chronological lifespan of *H. polymorpha.* Moreover, the possibility to generate NADH at carbon starvation conditions by an organic nitrogen source supports further extension of the lifespan of the cell. Consequently, the interpretation of CLS analyses in yeast should include possible effects on the energy status of the cell.

## Introduction

Aging is a degenerative process characterized by a progressive deterioration of cellular components resulting in enhanced mortality. Short lived model organisms, such as yeast, have strongly contributed to our current understanding of the molecular determinants of aging [1]. In yeast, two types of lifespan can be discriminated, referred to as replicative lifespan (RLS) and chronological lifespan (CLS). RLS is defined by the number of daughter cells a mother cell can produce before cell division ceases [2], whereas CLS is the time cells survive in the stationary phase [3]. These two types of lifespan can serve as models for proliferating (mitotic) and non-proliferating (post-mitotic) tissues in higher eukaryotes, respectively [4]. Research on CLS of glucose-grown *Saccharomyces cerevisiae* cells have strongly contributed to the identification of factors that contribute to aging. Using deletion or overexpression strains, several proteins have been identified that either negatively or positively influence CLS. For instance, deletion of certain genes involved in nutrient adaptation response, like *TOR1*, lengthens the lifespan of *S. cerevisiae* [5], [6], whereas the deletion of genes required for autophagy results in a reduced lifespan. In addition to genetic factors, also growth conditions have been shown to have major impact on the CLS of *S. cerevisiae* [7], [8]. Important parameters include the composition of the growth medium as well as the pH [9]. Finally, the addition of various compounds such as spermidine, resveratrol and rapamycin were demonstrated to have a positive effect on the CLS of *S. cerevisiae* [10], [11]. Both spermidine and rapamycin stimulate autophagy, underlining the importance of this process for longevity.

For several factors that affect aging in *S. cerevisiae* the molecular mechanisms have been elucidated in detail and shown to be conserved in higher eukaryotes. However, for many others the mechanisms are unclear or even highly debated.

Although *S. cerevisiae* is a highly attractive model organism for aging research, because of the unprecedented availability of tools and knowledge, this organism and the generally used growth substrate glucose, have also distinct disadvantages. First, *S. cerevisiae* is a Crabtree positive yeast, implying that mitochondrial respiration is turned down at high glucose conditions. As a result the organism shows diauxic growth on glucose: first glucose is consumed and converted into ethanol, followed by growth on ethanol. However, the metabolic intermediate ethanol (but also acetate that is formed as well) is an important determinant in CLS. Hence, a substrate that does not result in ethanol and acetate formation can have advantages in certain aging studies. Second, *S. cerevisiae* has lost several properties/genes during evolution that are still conserved from their common ancestor in other yeast species and animals. Finally, different from most yeast species, extensive gene duplication has occurred in *S. cerevisiae*, which requires the construction of double mutants in order to detect specific phenotypes. Indeed, the analysis of CLS in alternative yeast species, such as *Candida albicans, Kluyveromyces lactis* and *Schizosaccharomyces pombe*, has already been shown to contribute to the identification of universal molecular factors acting on aging [12].

In the present work, we have used the methylotrophic yeast species *Hansenula polymorpha* to study chronological aging. In contrast to *S. cerevisiae*, this yeast is Crabtree negative. This yeast also is capable to metabolize a range of carbon (e.g. methanol and ethanol) and organic nitrogen sources (primary amines, D-amino acids) that all require peroxisome function for growth which have not been analyzed in CLS studies before. Our data indicate that utilization of these compounds (ethanol, methanol, D-alanine, methylamine) results in enhanced CLS relative to glucose/ammonium sulphate.

## Materials and Methods

### Strain and growth conditions

*H. polymorpha* NCYC495 *leu1.1*, an amine oxidase deletion strain derived from this wild-type strain and *atg1* [13] were used throughout this study. Yeast cells were grown at 37°C on mineral medium (MM) [14] supplemented with different carbon sources (0.5% glucose, 0.5% methanol and 0.35% ethanol) and nitrogen sources (0.0025%, 0.25% MA and 0.33% D-alanine), unless stated otherwise. Leucine was added to a final concentration of 30 µg/ml. For viability determination cells were plated on YPD agar plates containing 1% yeast extract, 1% peptone, 1% glucose and 2% agar. For cloning purposes, *E. coli* DH5α was used; cells were grown at 37°C on LB media supplemented with 100 µg/ml ampicillin or 50 µg/ml kanamycin when required.

### Chronological lifespan measurements

**Figure 1:**
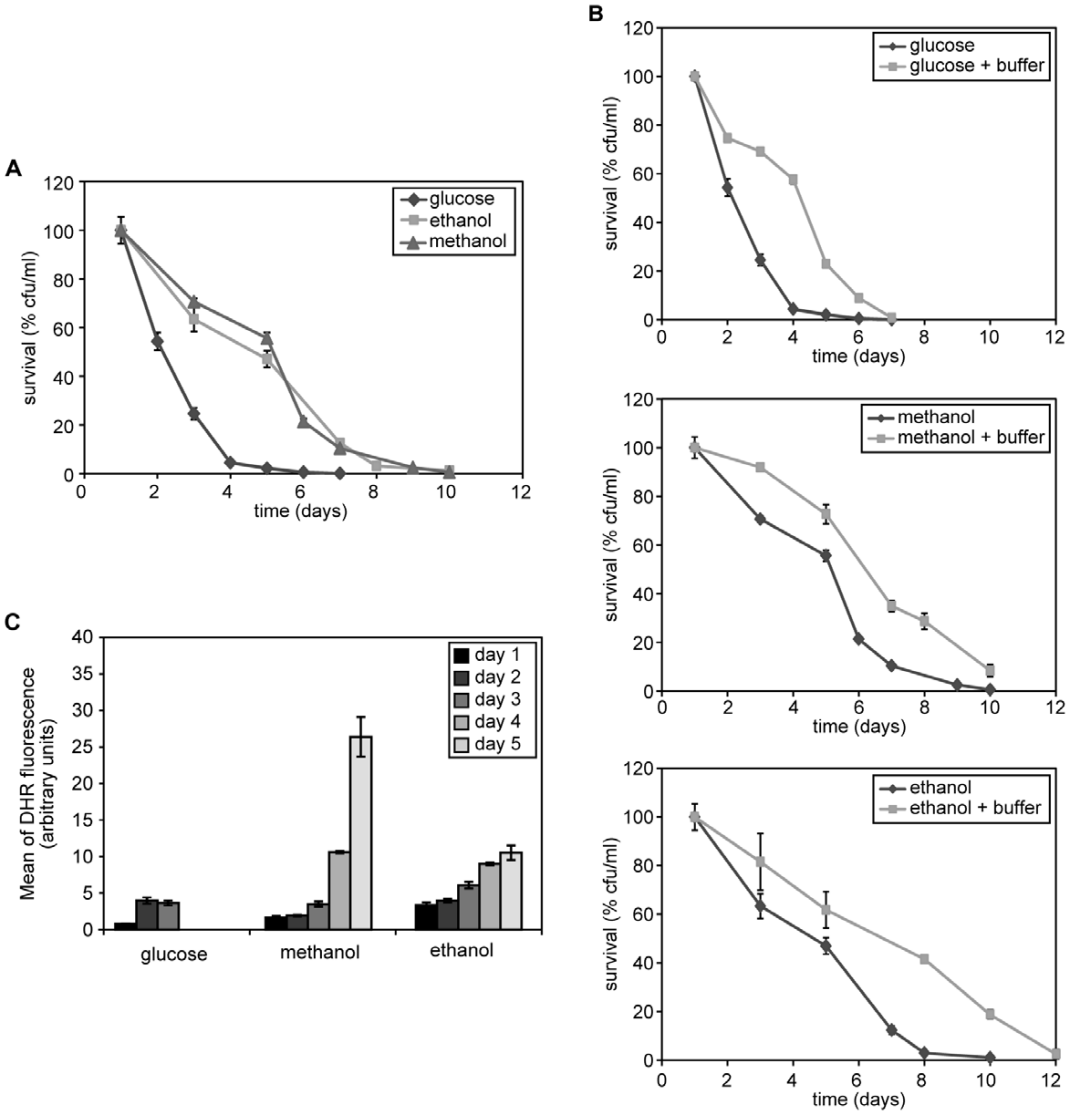
Chronological aging of *H. polymorpha* cells grown on different carbon sources. (A) CLS of wild-type cells following cultivation on various carbon sources (0.5% glucose, 0.35% ethanol or 0.5% methanol) in the presence of ammonium sulfate (AS) as nitrogen source. (B) CLS cultures grown like for panel A, but shifted to phosphate buffer pH 6 after reaching the stationary phase (16 h for glucose and 40 h for ethanol and methanol). (C) Measurement of DHR fluorescence in wild-type cells grown on different carbon sources as indicated in Figure 1A. Bars indicate the standard error of mean. The lifespan curves shown represent the average of 4–6 experiments.

Yeast cells from fresh YPD plates were inoculated into MM supplemented with 0.5% glucose and 0.25% ammonium sulfate and grown overnight. Overnight cultures were diluted to an OD_600 nm_ of 0.1 in the same medium and grown till OD_600 nm_ of 1.0 and again diluted 1/10 to the same medium. When the cultures reached an OD_600 nm_ of 1.5, cells were transferred to MM supplemented with different carbon and nitrogen sources at a start OD of 0.1. CLS measurements were started when the culture reached the stationary phase (16 h on glucose and 40 h on methanol and ethanol containing media) and was referred as day 1. Cells were kept in the spent medium except for the experiments shown in Fig. 1B, where stationary phase cells were collected by centrifugation and resuspended in 25 mM phosphate buffer pH 6.0. For viability assays, the number of cells per ml was determined using CASY® Model TT (Roche Applied Science). 500 cells were plated on YPD agar plates and incubated at 37°C until colonies appeared. The lifespan curves shown represent the average of 4–6 experiments.

### ROS measurements

ROS accumulation, mainly peroxides and peroxinitrites, was measured with dihydrorhodamine 123 (DHR, Invitrogen). 10^7^ cells were stained with 20 µg/ml DHR for 30 minutes. Mean of fluorescence was measured using a FACS Aria II Cell sorter (BD Biosciences) equipped with a 488 nm laser and 530/30 nm band-pass filter. FACSDiva software version 6.1.2 was used for data acquisition and analysis.

### Construction of Hansenula polymorpha Δamo mutant

The Δ*amo* strain was constructed by replacing the genomic region of *AMO* comprising nucleotides +103 to +1925 by the hygromycin B resistance gene, *HphMX4* in the wild-type cells. Two DNA fragments from −515 to +102 and +1926 to +2412 of the *AMO* genomic region were amplified by PCR using primers 5′Amo-FP (5′-GGGGACAACTTTGTATAGAAAAGTTGCTCAGCTTGGTGAGCACCTTCT-3′)/5′Amo-RP (5′-GGGGACTGCTTTTTTGTACAAACTTGCTTGATCTCGGCGGTGGACAG-3′) and 3′Amo-FP (5′-GGGGACAGCTTTCTTGTACAAAGTGGCTCTTAGACCTCGGCACTTCTT-3′)/3′Amo-RP (5′-GGGGACAACTTTGTATAATAAAGTTGCCAACAGACGACCTGATGAAC-3′) respectively, and *H. polymorpha* genomic DNA as a template. The PCR fragments were cloned into the vectors pDONR-P4-1R and pDONR-P2R-P3[15] , respectively, resulting in the entry vectors pENTR-AMO 5′ and pENTR-AMO 3′. Recombination of entry vectors pENTR-AMO 5′, pENTR-AMO 3′and pENTR-221-HPH [15] , and the destination vector pDEST-R4-R3 resulted in pSAN01. Subsequently, *H. polymorpha* WT *leu1.1* cells were transformed with the 2915 bp *amo::HphMX4* deletion fragment, which was obtained by PCR using primers Amo.cas.FP (5′-CAGCTTGGTGAGCACCTTCT-3′)/Amo.cas.RP (5′-CAACAGACGACCTGATGAAC-3′) and pSAN01 as a template. The resulting strain was designated as Δ*amo*. Correct integration was confirmed by PCR using primers EMK15 (5′- GAACTTCATTGACGCAGACGTC-3′)/Pex25-8PtefSc-Rv (5′GGGTGTTTTGAAGTGGTACG-3′) and AMO_del_RP (5′-CGAGTGGCGATGCAAACGAC-3′)/Pex25-10 TtefAg-Fw (5′-TCATCTGCCCAGATGCGAAG -3′).

### Biochemical methods

Yeast cells from aging cultures were harvested by centrifugation. Cell pellets were washed once with cold water and quickly frozen in liquid nitrogen; samples were stored at −80°C until further use. Cell extracts for enzyme activity measurements were prepared as described earlier [16]. AMO activity was measured as described previously [17]. Protein samples for SDS-PAGE gels were prepared and separated on 10% SDS-PAGE gel (BioRad). Proteins were transferred to nitrocellulose membranes using the semi-dry blotting method and probed with specific polyclonal anti-AMO antisera.

### Measurement of free amines in the culture medium

Supernatant from yeast cultures were collected upon spinning down the cells. Various dilutions were prepared in borate buffer (pH 9.0). One volume of fluorescamine solution (Sigma F9015., Saint Louis, Missouri, USA) was added to 1 ml of 100 to 1000 time diluted supernatant and mixed. Using a Fluoromax 3 spectrophotometer (Horiba, Kyoto, Japan) fluorescence intensity was measured (excitation at 390 nm; emission was collected from 400 nm to 600 nm). Fluorescence intensity, represented in count per second (cps) at 475 nm, was used for the calculations.

## Results

### Growth on ethanol or methanol extend the CLS relative to glucose

To investigate the effect of different carbon sources which are metabolized by peroxisome-borne enzymes on the CLS of *H. polymorpha*, we cultivated wild-type cells on mineral media containing ethanol or methanol as sole carbon source in the presence of ammonium sulphate (AS) as sole nitrogen source, using glucose/AS as control. The survival measurements started when the cells reached the stationary phase (day 1) and the cells were kept in their original medium. The data presented in Fig. 1A show that the CLS (both medium and maximum lifespan; Table 1) of the ethanol and methanol cultures was strongly extended relative to that of the glucose culture.

**Table 1:** The effect of buffer on median and maximum lifespan of *H. polymorpha*.

Carbon source*	Condition**	Median lifespan (days)	Maximum lifespan (days)
Glucose	Medium	2,2	3,8
Glucose	Buffer	4,2	5,9
Ethanol	Medium	4,6	7,3
Ethanol	Buffer	6,8	7.1
Methanol	Medium	5,1	7,9
Methanol	Buffer	6,2	10,0

* Cells were grown in the presence of ammonium sulphate as nitrogen source

** Upon reaching the stationary phase the cells were kept in the same medium or collected by centrifugation and resuspended in buffer.

The median lifespan is defined as time point when 50% of the cells survive, the maximum lifespan when 10% of the cells survive (Fig. 1A).

In *S. cerevisiae* acidification of the medium is an important factor in the CLS of glucose-grown cells. We therefore monitored whether differences occurred in the pH of the cultures during the CLS experiments. The pH of the glucose culture rapidly dropped from 6.2 to 3.8 during the first day of the experiment, whereas in the ethanol and methanol cultures the pH never dropped below 4.5.

To analyse whether medium acidification explained the reduced lifespan of the glucose cultures, CLS experiments were repeated using cells which were precultivated on the three different carbon sources and, upon reaching the stationary phase, collected by centrifugation and resuspended into phosphate buffer (pH 6). As shown in Fig. 1B and Table 1, the mean and maximum lifespan of the glucose-grown cells was strongly enhanced in phosphate buffer relative to the cultures that remained in the original medium. For the methanol and ethanol cultures the median and maximum lifespan only slightly increased. Hence, medium acidification is an important factor in the relatively short CLS of glucose-grown *H. polymorpha* cells, but plays a minor role in survival of methanol or ethanol cultures.

In addition to medium acidification, intracellular ROS levels are important in determining yeast CLS. We therefore analysed the levels of these reactive compounds in the three cultures using the dye DHR and FACS. As shown in Fig. 1C, glucose-grown cells accumulate similar, relatively low ROS levels like ethanol or methanol grown cells during the first 3days after reaching the stationary phase, when large differences in survival were already apparent (compare Fig. 1A). At later time points (day 4 and 5) we only measured ROS in methanol and ethanol cultures as most cells of the glucose culture already had died after day 3. On days 4 and 5 ROS levels increased in both cultures. The highest levels were observed in methanol-grown cells even though the survival of these cells was similar to ethanol-grown cells (Fig. 1A). These findings suggest that growth of cells on media that require peroxisome function has a positive effect on their despite the enhanced ROS levels.

### Methylamine extends the lifespan

**Figure 2:**
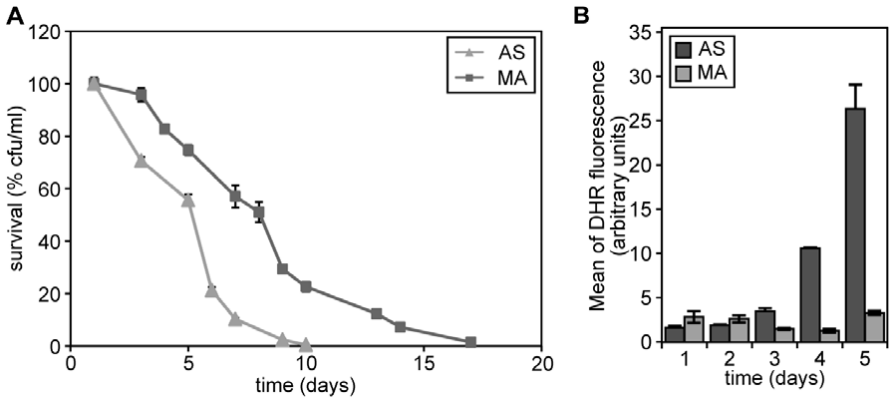
Chronological aging of *H. polymorpha* cells grown on different nitrogen sources. (A) CLS of wild-type cells following cultivation on 0.5% methanol in the presence of 0.25% AS or 0.25% MA s sole nitrogen sources. The lifespan curves shown represent the average of 4–6 experiments. (B) DHR fluorescence in cells grown on methanol in the presence of AS or MA at different time points during chronological aging. The bars indicate the standard error of mean of two independent experiments.

Different from *S. cerevisiae, H. polymorpha* is capable to utilize a large range of organic nitrogen sources. To test the effect of an organic nitrogen source on chronological aging, cells were grown on methanol in the presence of AS or methylamine (MA). As shown in Figure 2A, MA significantly extends the median and maximum lifespan relative to AS. In both the methanol/AS and methanol/MA cultures the pH had decreased only slightly (to 5.2) at day 1. After this time point the pH of both cultures remained constant suggesting that differences in acidification do not explain the observed CLS extension by MA.

We also measured ROS levels in both cultures. The data (Fig. 2B) revealed that during the first 3 days, when differences in survival were evident, ROS levels were at a similar, low level in both cultures. At days 4 and 5 the ROS levels remained low in the MA cultures whereas they increased in the AS cultures.

These data indicate that during the first days of the CLS experiment neither reduced acidification nor altered ROS levels can explain the positive effect of the utilization of MA as nitrogen source on the chronological lifespan. Hence, it is likely that additional processes play an important factor in the lifespan extension by MA.

### The CLS extension by MA is not dependent on autophagy

**Figure 3:**
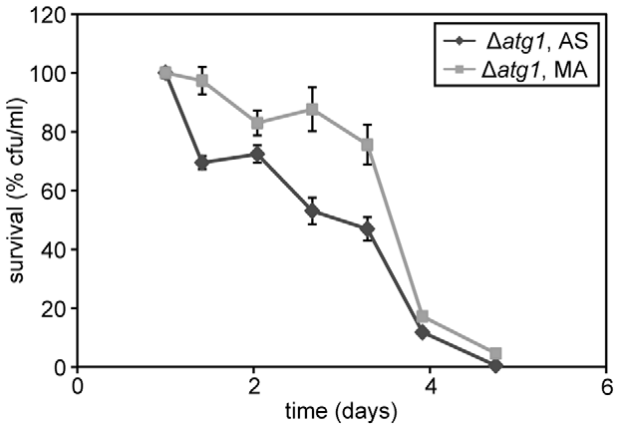
Chronological aging of *H. polymorpha* Δ*atg1* cells. CLS of Δ*atg1* cells grown on methanol in the presence of AS or MA. Bars indicate the standard error of mean of four experiments.

Autophagy has been shown to be important for yeast chronological aging. The polyamine spermidine prolongs the CLS in *S. cerevisiae* by inducing autophagy [11]. We therefore asked whether the lifespan extension caused by MA is related to changes in autophagy. If alterations in autophagy would explain the lifespan extension by MA, this extension should not occur in cells defective in autophagy. We therefore performed a CLS experiment using *H. polymorpha* Δ*atg1* cells, which are deficient in autophagy. These experiments indicated that the CLS of *H. polymorpha* Δ*atg1* cells is strongly reduced relative to that of the wild-type control (Fig. 3, compare also Fig. 1A). However, also in Δ*atg1* cultures a positive effect of MA on survival was evident during the first three days. In addition a slight increase in median and maximum lifespan was observed (Fig. 3). This result suggests that the use of MA has a positive effect on cell survival in an autophagy-independent way.

### The extended CLS is dependent on the MA concentration in the growth medium

**Figure 4:**
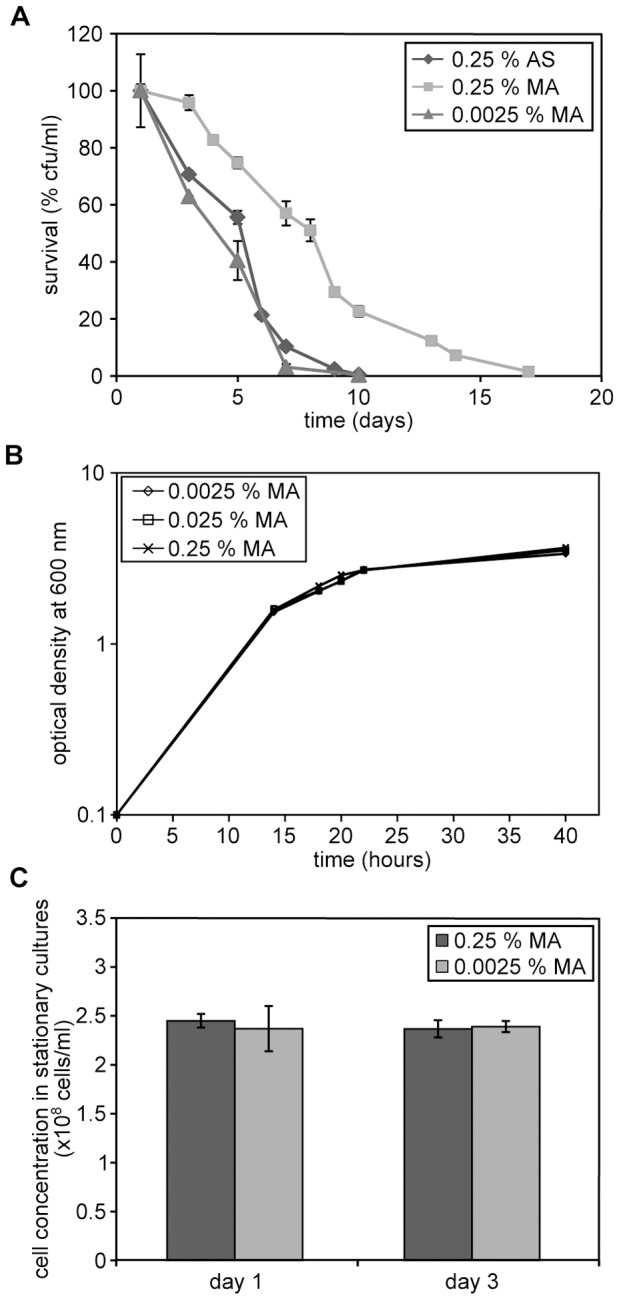
CLS extension relates to MA concentrations in the cultivation media. (A) CLS of wild-type cells grown on methanol in the presence of 0.25% ammonium sulphate (AS) or 0.25% or 0.0025% MA. Bars indicate the standard error of mean of four experiments (B) Growth curves of wild-type *H. polymorpha* grown on methanol in the presence of different concentrations of MA (0.25%, 0.025% or 0.0025%). Optical densities are expressed as absorption at 600 nm. (C) Cell concentrations expressed as number of cells per ml in stationary cultures grown on media containing 0.25% or 0.0025% MA.

**Figure 5:**
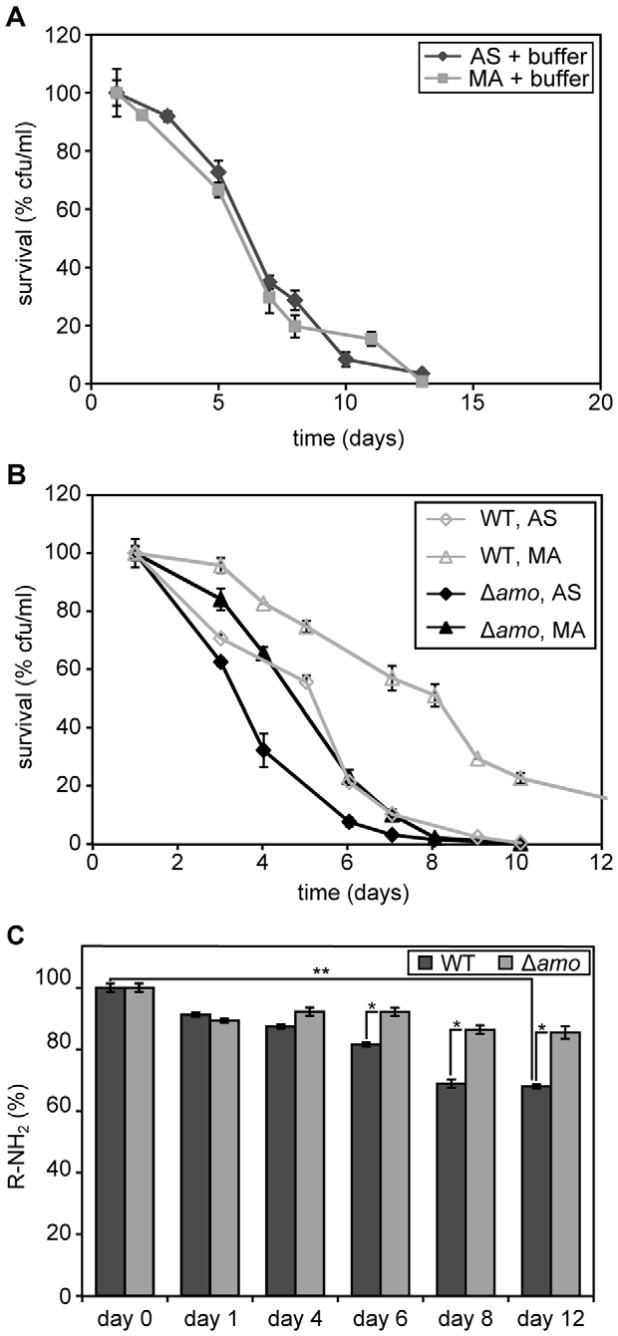
MA metabolism is required for CLS extension. (A) CLS of cells which were pregrown on methanol/AS or methanol/MA until the stationary phase and subsequently harvested and resuspended in phosphate buffer. (B) CLS of wild-type and Δ*amo* cultures grown on methanol in the presence of 0.25% MA or 0.25% AS. Bars indicate the standard error of mean of two independent experiments. (C) Levels of free amines (-NH_2_) in the cultivation media of cultures of wild-type and Δ*amo* cells grown on methanol in the presence of 0.25% MA. The level of free amines in medium containing 0.25% MA before inoculation was set to 100%. Bars indicate the standard error of mean of two independent experiments. Statistical analysis was performed by student t-test, * = p<0.05; ** = p<0.01.

Like spermidine, MA may directly trigger specific cellular processes that contribute to cell survival. Alternatively, MA metabolism may be responsible for the observed lifespan extension. In the latter case it is likely that the positive effect of MA is only observed at relatively high concentrations. To test this, we analysed the effect of reducing the concentration of MA. No change in the CLS curve was observed (relative to the methanol/AS) when the MA concentration was 100 fold reduced (Fig. 4A). This was not due to negative effects of the low MA concentration on the initial growth phase as both the doubling time (Fig. 4B) and the final yield of the culture (Fig. 4C) was unaltered at reduced MA concentrations. This suggests that the metabolism of MA during the stationary phase is important for lifespan extension. If so, the presence of MA in the medium should be essential to extend the CLS. To analyse this, we removed MA from the stationary phase cultures. To this end cells were precultivated on methanol/AS or methanol/MA until the stationary phase. Subsequently both cultures were harvested by centrifugation and resuspended in phosphate buffer (pH 6.0). As shown in Figure 5A, methanol/AS and methanol/MA grown cells showed a very similar CLS curve in buffer.

When MA metabolism is responsible for the CLS extension, this effect should be abolished in a strain lacking amine oxidase (AMO) activity. Indeed, MA did not result in a CLS extension in an AMO deficient strain (Δ*amo* Fig. 5B).

Finally, if MA consumption is important the levels of free amines in the media should drop during chronological aging and the cells should display AMO activity in the stationary phase. Measurement of free amine levels (Fig. 5C) revealed a gradual decreased, indicating that MA is consumed during the initial growth but also during the stationary phase. As expected, no significant decline was observed in cultures of the Δ*amo* control strain.

**Figure 6:**
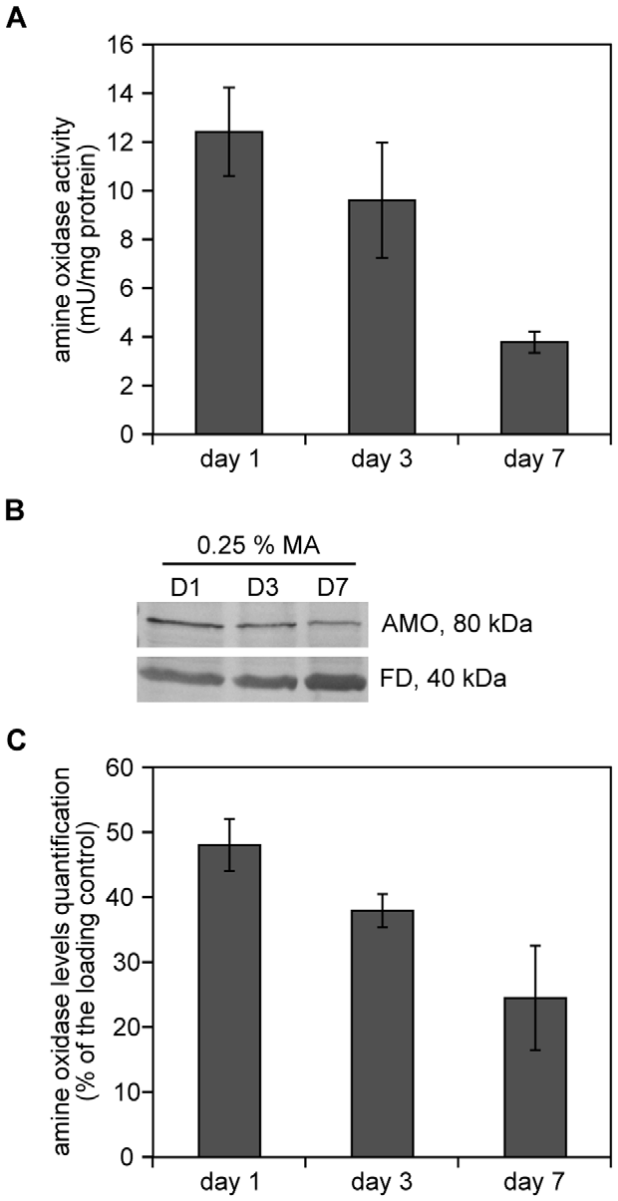
Specific AMO activities and AMO protein levels decrease during chronological aging. (A) Detection of AMO activities in wild-type cells in cultures grown on methanol in the presence of 0.25% methylamine during chronological aging. Bars indicate the standard error of mean of two independent experiments. (B) Western blot analysis of AMO protein levels in wild-type cells grown in the presence of 0.25% MA or on 0.25% ammonium sulphate (AS). Blots were decorated with specific antibodies against AMO. Formate dehydrogenase (FD) was used as loading control. (C) Quantification of the AMO levels using densitometric scanning of the blots. Two independent blots were quantified. The error bars indicate the standard error. The loading control was set to 100%.

Enzyme assays revealed that AMO activity was present during the CLS experiment, but somewhat reduced after 7 days relative to the values observed at day 1 and 3 (Fig. 6A). The reduction in AMO activity at day 7 was paralleled by a reduction in AMO protein as was observed by Western blot experiments (Fig. 6B, C).

### Formaldehyde can cause an increase in CLS similar as MA

**Figure 7:**
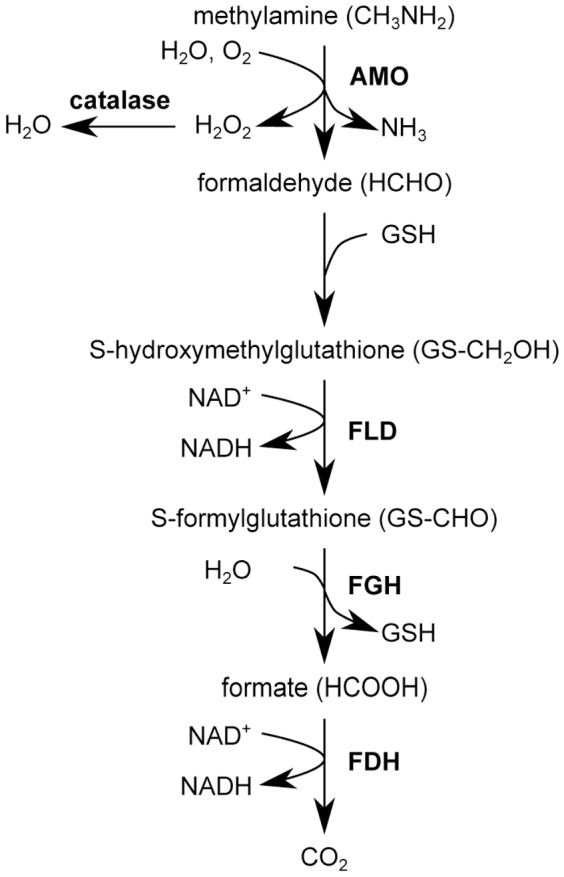
Schematic overview of MA metabolism in *H. polymorpha*. MA is oxidized by peroxisomal amine oxidase (AMO) to generate formaldehyde, ammonium and hydrogen peroxide. After binding of glutathione (GSH) to formaldehyde, the produced S-hydroxymethylglutathione is converted to S-formylglutathione by formaldehyde dehydrogenase (FLD). GSH is removed by S-formyl glutathione hydrolase (FGH) and formate is converted to CO_2_ by formate dehydrogenase (FDH). Oxidation of formaldehyde generates 2 molecules of NADH that are used for ATP generation in mitochondria [30].

**Figure 8:**
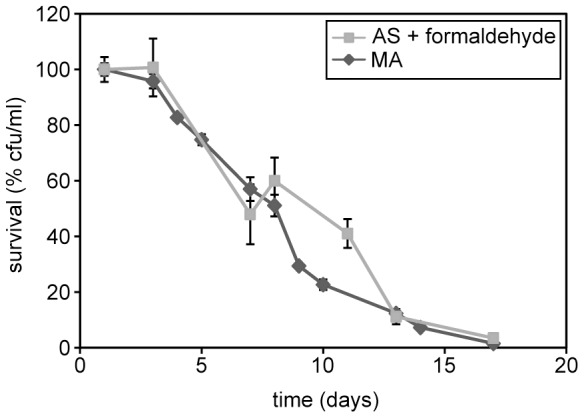
Formaldehyde extends the CLS. Wild-type cells were grown on methanol media containing 0.25% ammonium sulphate or 0.25% MA. Upon reaching the stationary phase, the ammonium sulphate containing culture was supplemented with 37.8 mM formaldehyde. The MA cultures were kept in the same medium as a control. Bars indicate the standard error of mean of 2 experiments.

MA is oxidized by AMO into ammonium and formaldehyde [17]. In *H. polymorpha* formaldehyde is further oxidized by formaldehyde dehydrogenase and formate dehydrogenase into CO_2_. This process results in the generation of 2 NADH molecules (Fig. 7). To test whether the formaldehyde oxidation product of MA was responsible for the extension of the CLS by MA, we supplemented a methanol/AS grown stationary phase culture with 37.8 mM formaldehyde, using cultures with 37.8 mM MA (corresponding to 0.25% w/v) as a control. The data revealed that the chronological lifespan of cultures supplemented with formaldehyde is extended to the same extent as MA cultures (Fig. 8). These data suggest that oxidation of formaldehyde, generated by MA oxidation during the stationary phase, is likely responsible for extra NADH supply which leads to an enhanced lifespan.

### D-alanine results in lifespan extension

**Figure 9:**
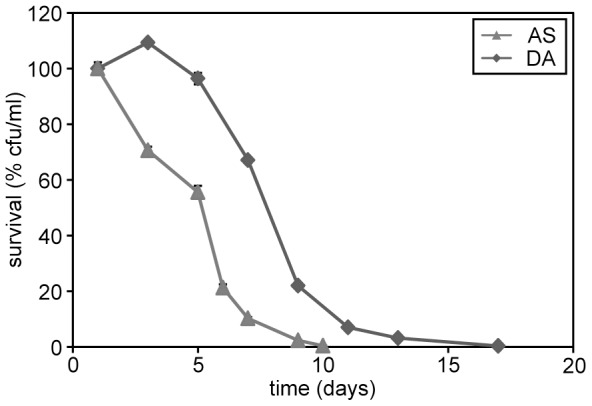
Chronological aging of *H. polymorpha* cells grown on D-alanine. CLS of wild-type cells following cultivation on 0.5% methanol in the presence of 0.25% AS or 0.33% D-alanine (DA) as sole nitrogen sources. The lifespan curves shown represent the average of 4–6 experiments.

*H. polymorpha* can also use D-alanine as nitrogen source. If energy generation during the stationary phase can extend the lifespan of cells, D-alanine is expected to cause a similar effect as MA. D-alanine is oxidized by D-amino acid oxidase into ammonium and pyruvate [18]. As shown in Fig. 9, indeed D-alanine prolongs the chronological lifespan of *H. polymorpha*.

## Discussion

Yeast chronological aging has a multifactorial nature. Many cellular processes and extrinsic factors negatively influence the CLS. Examples include oxidative stress, reduced autophagy or medium acidification. Processes which induce stress responsive genes extend yeast lifespan [19] . Hence yeast CLS is determined by the resultant of multiple positive and negative processes. Because of this complexity several factors implicated in yeast aging are still highly debated.

So far most research on yeast CLS is performed with *S. cerevisiae* using glucose/ammonium sulphate containing media. In this paper we analysed the chronological lifespan of the yeast *H. polymorpha* in relation to growth on different carbon and nitrogen sources.

Our data revealed that of the three carbon sources tested (glucose relative to two compounds that require peroxisome function for growth namely ethanol and methanol) *H. polymorpha* shows the shortest chronological lifespan on glucose. Compared to *S. cerevisiae*, *H. polymorpha* dies relatively fast with a short maximum lifespan of less than 4 days when grown on 0.5% glucose. At these conditions the maximum lifespan of *S. cerevisiae* is generally above 10 days [20] .

Glucose metabolism involves glycolysis, which in *S. cerevisiae* leads to acetic acid formation that is associated with induction of the mitochondrial apoptosis pathway [21]. In *S. cerevisiae* acetic acid production is strongly reduced upon growth on glycerol instead of glucose [22] (. Similar mechanisms most likely operate in *H. polymorpha*, because the medium of glucose cultures acidified more strongly relative to those containing ethanol- or methanol. Although placing the glucose-grown cells in fresh buffer significantly extended the lifespan, neither the median nor maximum lifespan reached values obtained for methanol or ethanol cultures (Table 1). One explanation may be that prior to placing the glucose-grown cells in buffer, they already experienced the toxic effects related to the low pH when reaching the stationary phase.

In addition to medium acidification, ROS are important factors in determining yeast CLS. ROS initially were assumed to be harmful as they caused oxidative damage. However, data have been presented indicating that ROS also can have a positive effect as signaling molecules that induce stress responsive genes (hormesis). Moreover, recent findings suggest that in *S. cerevisiae* also the type of ROS (e.g. superoxide versus hydrogen peroxide) and the growth stage at which they occur are important for lifespan extension [23], [24], which illustrates a complex role of ROS in yeast aging.

We observed that in *H. polymorpha* ROS levels were equally low during the first days of the CLS experiments in glucose, ethanol and methanol cultures. Because in this period differences in survival were already evident, ROS levels alone most likely are not the major determinants in the observed differences in lifespan. At later stages ROS levels increased in the methanol and ethanol cultures. Given the multifactorial nature of CLS, it is yet unclear whether this may have caused negative and/or positive effects. Also, ROS measurements performed with fluorescent dyes have to be interpreted with care when cells are grown on different carbon sources. We used DHR, which forms fluorescent rhodamine efficiently upon reaction with free ^•^OH or NO_2_^•^ radicals, but requires a catalyst for oxidation by O_2_^•^ or H_2_O_2_ [25]. Important catalysts are iron, heme and cytochrome c oxidase. These catalysts as well as the composition of the ROS may vary significantly upon growth of *H. polymorpha* cells on the different carbon sources. For instance, the peroxisomal heme containing enzyme catalase is strongly induced on methanol, to moderate levels on ethanol but repressed on glucose [26]. Hence, relative to glucose heme levels and most likely also iron (e.g. released from catalase in aged cells) are may be significantly higher in methanol and ethanol cells which may add to the observed increase in ROS levels. Together, our data lend support to the view that cultivation of cells at conditions that require peroxisomes for growth is beneficial for the lifespan of the cells.

It cannot be excluded that other factors also contribute to the short CLS of glucose-grown *H. polymorpha*. For instance, by-products of glycolysis like methylglyoxal were described to have a negative impact on cell survival [27]. Further studies are required to fully dissect all factors involved.

Our data indicate that the presence of methylamine (MA) as sole nitrogen source instead of ammonium sulphate resulted in a significant extension of the chronological lifespan of methanol-grown cells. Because no differences in pH values were observed, medium acidification is not a major factor in the observed lifespan differences.

Amines have been described to universally enhance the lifespan of various models. For instance, spermidine acts as an anti-aging compound by inducing autophagy [11]. Because we also observed the positive effect of MA on viability in *H. polymorpha* Δ*atg1* cells that are defective in autophagy (Fig. 3), MA is unlikely to alter autophagy processes in this yeast species. Moreover, based on electron microscopy studies we did not obtain any morphological indications that MA induces or reduces autophagy in wild-type cells (data not shown).

Our studies indicate that MA oxidation by AMO and the subsequent generation of extra NADH, is an important reason for the lifespan extension by MA. We show that the positive effects of MA do not occur in an AMO deficient strain or when MA is removed from the medium, but occur again when formaldehyde, the oxidation product of MA, was added to the stationary phase cultures. This led us to conclude that the generation of additional NADH in carbon starved cells can postpone cell death by providing energy and reducing the intracellular environment. Indeed, our data (Fig. 2B) suggest that NADH generation due to methylamine metabolism changes the intracellular redox balance in the cell leading to lower ROS levels. Hence, the relatively high ROS levels observed in methanol/AS cultures (see also Fig. 1C) might present a detrimental effect.

Similar to MA, D-alanine can also be used as a nitrogen source by *H. polymorpha*. Oxidative deamination of D-alanine generates pyruvate and ammonia. We anticipate that production of pyruvate during chronological aging generates energy and extends the CLS in similar fashion as MA [18], [28].

Summarizing, our data are consistent with the view that multiple factors may be involved in lifespan extension caused by MA. Our data indicate that production of NADH generated from MA metabolism is the major factor. NADH contributes to ATP generation but also to reducing ROS. Additional factors may be involved as well, such as toxicity of ammonium in methanol/AS cultures, like recently reported for *S. cerevisiae* [29].).

Chronological aging of yeast cells has been proposed as a model for the post-mitotic cells in higher eukaryotes [19], a situation which is obviously dissimilar to starving cells in yeast stationary phase cultures. However, the use of an additional NADH generation system, which does not support growth, can make findings of yeast systems more applicable as model for higher eukaryotic cells.

## References

[1] Kennedy BK (2008) The genetics of ageing: insight from genome-wide approaches in invertebrate model organisms. J Intern Med 263: 142–152. 18226092 10.1111/j.1365-2796.2007.01903.x

[2] Kennedy BK, Austriaco NR Jr, Guarente L (1994) Daughter cells of *Saccharomyces cerevisiae* from old mothers display a reduced life span. J Cell Biol 127: 1985–1993. 7806576 10.1083/jcb.127.6.1985 PMC2120297

[3] MacLean M, Harris N, Piper PW (2001) Chronological lifespan of stationary phase yeast cells; a model for investigating the factors that might influence the ageing of postmitotic tissues in higher organisms. Yeast 18: 499–509. 11284006 10.1002/yea.701

[4] Kaeberlein M (2010) Lessons on longevity from budding yeast. Nature 464: 513–519. 20336133 10.1038/nature08981 PMC3696189

[5] Fabrizio P, Pozza F, Pletcher SD, Gendron CM, Longo VD (2001) Regulation of longevity and stress resistance by Sch9 in yeast. Science 292: 288–290. 11292860 10.1126/science.1059497

[6] Roosen J, Engelen K, Marchal K, Mathys J, Griffioen G, et al (2005) PKA and Sch9 control a molecular switch important for the proper adaptation to nutrient availability. Mol Microbiol 55: 862–880. 15661010 10.1111/j.1365-2958.2004.04429.x

[7] Alvers AL, Fishwick LK, Wood MS, Hu D, Chung HS, et al (2009) Autophagy and amino acid homeostasis are required for chronological longevity in *Saccharomyces cerevisiae* . Aging Cell 8: 353–369. 19302372 10.1111/j.1474-9726.2009.00469.x PMC2802268

[8] Masoro EJ (2005) Overview of caloric restriction and ageing. Mech Ageing Dev 126: 913–922. 15885745 10.1016/j.mad.2005.03.012

[9] Piper PW, Breitenbach M, Jazwinski SM, Laun P (2012) Maximising the yeast chronological lifespan. In: Breitenbach M, Jazwinski SM, Laun P, editors. Aging Research in Yeast: Springer Science+Business Media. pp. 145–159.

[10] Morselli E, Mariño G, Bennetzen MV, Eisenberg T, Megalou E, et al (2011) Spermidine and resveratrol induce autophagy by distinct pathways converging on the acetylproteome. J Cell Biol 192: 615–629. 21339330 10.1083/jcb.201008167 PMC3044119

[11] Eisenberg T, Knauer H, Schauer A, Buttner S, Ruckenstuhl C, et al (2009) Induction of autophagy by spermidine promotes longevity. Nat Cell Biol 11: 1305–1314. 19801973 10.1038/ncb1975

[12] Roux AE, Chartrand P, Ferbeyre G, Rokeach LA (2010) Fission yeast and other yeasts as emergent models to unravel cellular aging in eukaryotes. The Journals of Gerontology Series A: Biological Sciences and Medical Sciences 65A: 1–8. 10.1093/gerona/glp152 19875745

[13] Komduur JA, Veenhuis M, Kiel JA (2003) The *Hansenula polymorpha PDD7* gene is essential for macropexophagy and microautophagy. FEMS Yeast Res 3: 27–34. 12702243 10.1016/s1567-1356(02)00135-6

[14] Dijken LP, Otto R, Harder W (1976) Growth of *Hansenula polymorpha* in a methanol-limited chemostat. Arch Microbiol 111: 137–144. 1015956 10.1007/BF00446560

[15] Saraya R, Krikken AM, Kiel JAKW, Baerends RJS, Veenhuis M, et al (2012) Novel genetic tools for *Hansenula polymorpha* . FEMS Yeast Research 12: 271–278. 22129301 10.1111/j.1567-1364.2011.00772.x

[16] Waterham HR, Keizer-Gunnink I, Goodman JM, Harder W, Veenhuis M (1992) Development of multipurpose peroxisomes in *Candida boidinii* grown in oleic acid-methanol limited continuous cultures. J Bacteriol 174: 4057–4063. 1350779 10.1128/jb.174.12.4057-4063.1992 PMC206116

[17] Zwart K, Veenhuis M, Dijken JP, Harder W (1980) Development of amine oxidase-containing peroxisomes in yeasts during growth on glucose in the presence of methylamine as the sole source of nitrogen. Arch Microbiol 126: 117–126. 7192080 10.1007/BF00511216

[18] Pollegioni L, Piubelli L, Sacchi S, Pilone M, Molla G (2007) Physiological functions of D-amino acid oxidases: from yeast to humans. Cell Mol Life Sci 64: 1373–1394. 17396222 10.1007/s00018-007-6558-4 PMC11136250

[19] Longo Valter D, Shadel Gerald S, Kaeberlein M, Kennedy B (2012) Replicative and chronological aging in *Saccharomyces cerevisiae* . Cell metabolism 16: 18–31. 22768836 10.1016/j.cmet.2012.06.002 PMC3392685

[20] Smith JDL, McClure JM, Matecic M, Smith JS (2007) Calorie restriction extends the chronological lifespan of *Saccharomyces cerevisiae* independently of the Sirtuins. Aging Cell 6: 649–662. 17711561 10.1111/j.1474-9726.2007.00326.x

[21] Ludovico P, Rodrigues F, Almeida A, Silva MT, Barrientos A, et al (2002) Cytochrome c release and mitochondria involvement in programmed cell death induced by acetic acid in *Saccharomyces cerevisiae* . Molecular Biology of the Cell 13: 2598–2606. 12181332 10.1091/mbc.E01-12-0161 PMC117928

[22] Burtner CR, Murakami CJ, Kennedy BK, Kaeberlein M (2009) A molecular mechanism of chronological aging in yeast. Cell Cycle 8: 1256–1270. 19305133 10.4161/cc.8.8.8287 PMC2746416

[23] Mesquita A, Weinberger M, Silva A, Sampaio-Marques B, Almeida B, et al (2010) Caloric restriction or catalase inactivation extends yeast chronological lifespan by inducing H_2_O_2_ and superoxide dismutase activity. Proceedings of the National Academy of Sciences 107: 15123–15128. 10.1073/pnas.1004432107 PMC2930563 20696905

[24] Pan Y, Schroeder EA, Ocampo A, Barrientos A, Shadel GS (2011) Regulation of Yeast Chronological Life Span by TORC1 via Adaptive Mitochondrial ROS Signaling. Cell metabolism 13: 668–678. 21641548 10.1016/j.cmet.2011.03.018 PMC3110654

[25] Wardman P (2007) Fluorescent and luminescent probes for measurement of oxidative and nitrosative species in cells and tissues: Progress, pitfalls, and prospects. Free Radical Biology and Medicine 43: 995–1022. 17761297 10.1016/j.freeradbiomed.2007.06.026

[26] van Dijken JP, Veenhuis M, Vermeulen CA, Harder W (1975) Cytochemical localization of catalase activity in methanol-grown *Hansenula polymorpha* . Arch Microbiol 105: 261–267. 53039 10.1007/BF00447145

[27] Hipkiss AR (2010) Mitochondrial dysfunction, proteotoxicity, and aging: causes or effects, and the possible impact of NAD^+^-controlled protein glycation. Adv Clin Chem 50: 123–150. 20521444

[28] Sulter GJ, Waterham HR, Goodman JM, Veenhuis M (1990) Proliferation and metabolic significance of peroxisomes in *Candida boidinii* during growth on D-alanine or oleic acid as the sole carbon source. Arch Microbiol 153: 485–489. 2339955 10.1007/BF00248431

[29] Santos J, Sousa MJ, Leão C (2012) Ammonium Is Toxic for Aging Yeast Cells, Inducing Death and Shortening of the Chronological Lifespan. PLoS ONE 7: e37090. 22615903 10.1371/journal.pone.0037090 PMC3352862

[30] Van Dijken JP, Harder W, Beardsmore AJ, Quayle JR (1978) Dihydroxyacetone: An intermediate in the assimilation of methanol by yeasts? FEMS Microbiology Letters 4: 97–102.

